# Attention-based dual-path feature fusion network for automatic skin lesion segmentation

**DOI:** 10.1186/s13040-023-00345-x

**Published:** 2023-10-09

**Authors:** Zhenxiang He, Xiaoxia Li, Yuling Chen, Nianzu Lv, Yong Cai

**Affiliations:** 1 School of Information Engineering, Southwest University of Science and Technology, Mianyang, China; 2 Tianfu College of Southwest University of Finance and Economics, Mianyang, China; 3 Robot Technology Used for Special Environment Key Laboratory of Sichuan Province, Mianyang, China; 4 School of manufacturing science and Engineering, Southwest University of Science and Technology, Mianyang, China

**Keywords:** Skin lesion segmentation, Convolution neural network, Boundary refinement

## Abstract

Automatic segmentation of skin lesions is a critical step in Computer Aided Diagnosis (CAD) of melanoma. However, due to the blurring of the lesion boundary, uneven color distribution, and low image contrast, resulting in poor segmentation result. Aiming at the problem of difficult segmentation of skin lesions, this paper proposes an Attention-based Dual-path Feature Fusion Network (ADFFNet) for automatic skin lesion segmentation. Firstly, in the spatial path, a Boundary Refinement (BR) module is designed for the output of low-level features to filter out irrelevant background information and retain more boundary details of the lesion area. Secondly, in the context path, a Multi-scale Feature Selection (MFS) module is constructed for high-level feature output to capture multi-scale context information and use the attention mechanism to filter out redundant semantic information. Finally, we design a Dual-path Feature Fusion (DFF) module, which uses high-level global attention information to guide the step-by-step fusion of high-level semantic features and low-level detail features, which is beneficial to restore image detail information and further improve the pixel-level segmentation accuracy of skin lesion. In the experiment, the ISIC 2018 and PH2 datasets are employed to evaluate the effectiveness of the proposed method. It achieves a performance of 0.890/ 0.925 and 0.933 /0.954 on the F1-score and SE index, respectively. Comparative analysis with state-of-the-art segmentation methods reveals that the ADFFNet algorithm exhibits superior segmentation performance.

## Introduction

**Fig. 1:**
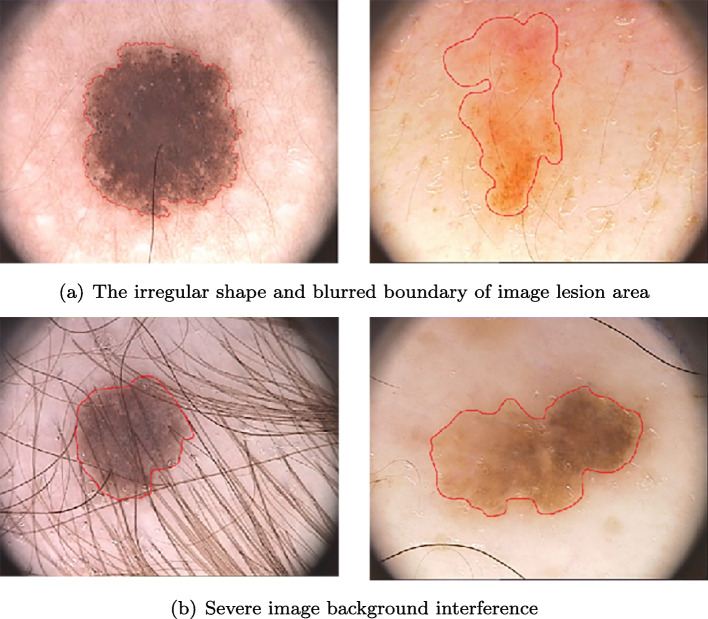
The main challenges of segmentation of melanoma (the red outline indicates the lesion area, and images are from the PH2 dataset)

Skin cancer has become one of the most serious public health problems due to its high rates of morbidity and mortality. Among them, melanoma is the deadliest of all skin cancers, and 75% of skin cancer patients die as a result [1, 2]. Studies have shown that when melanoma is limited to the outer layer of the skin, simple resection is usually curable. The cure rate of early patients is as high as 95% [3], but unfortunately, many patients are diagnosed as more advanced and incurable, so early screening is extremely necessary. Dermoscopy image analysis plays an important role in the early detection of melanoma, but it takes a long time to manually screen the image by dermatologists and is easily affected by subjective experience. In recent years, Computer Aided Diagnosis (CAD) systems have been increasingly used in the diagnosis of patients with skin diseases [4]. A vision-based Automated CAD system includes five main steps: image acquisition, data processing, lesion segmentation, feature extraction, and lesion recognition, among which lesion segmentation is a key step in automated CAD systems and subsequent treatment. Skin lesion segmentation refers to the pixel-by-pixel classification of dermoscopy images to delimit the boundaries of the lesion, thereby separating diseased skin from healthy skin. The quality of skin lesion segmentation has an important influence on the accuracy of the results of the CAD systems. Image detection method is used to determine whether skin lesions are normal, benign or malignant [5]. The skin lesion segmentation algorithm is used to automatically obtain the accurate lesion area in the dermoscopy image, which can greatly facilitate a doctor’s analysis and evaluation of the clinical characteristics of the lesion area, which can effectively improve the accuracy of early diagnosis of melanoma and reduce the rate of misdiagnosis and missed diagnosis. However, using segmentation algorithms to automatically obtain accurate lesion areas in the dermoscopy image still faces the following two challenges. First, the skin pigmented lesions have irregular shapes and blurred borders, making it difficult to finely segment the borders. Secondly, uncontrollable background factors such as skin color, skin surface hair, capillaries, and bubbles will seriously affect the effect of lesion area segmentation. We illustrate these challenges through some examples in Fig. 1.

A lot of efforts have been dedicated to solve these challenges. In early research, the segmentation of skin lesions was mainly based on classic digital image segmentation methods [6], including histogram threshold segmentation [7–9], edge detection [10], region growth method [11, 12], and active contour model [13] and so on. The histogram threshold segmentation method is relatively simple, but due to the irregular boundary of the lesion area and uneven color distribution in the skin pigmented lesion image, it is difficult to find a suitable threshold for segmentation. The edge detection method uses different gradient operators to extract the edge of the lesion area. However, due to the low contrast between the part of the lesion area and the normal skin area, the boundary is blurred, and the background interference is serious will cause under-segmentation or segmentation failure. The region growing method is iterative, and the noise and uneven gray level of the skin lesions are likely to cause over-segmentation. The active contour model method is more sensitive to noise and will fail to segment when there is interference from hair, blood vessels, and bubbles in the skin lesion area.

In recent years, Convolution Neural Network (CNN) [14] has been widely used in the field of target detection and segmentation with its powerful feature extraction capabilities. For the medical image segmentation problem, the early approach was to slice the image by sliding window, and then predict whether each block is in the background area or the target area through the CNN network, and convert the segmentation problem into a pixel classification problem [15, 16], but there is a lot of excessive calculation while failing to effectively use the image context information, which has certain limitations. As Long et al. proposed a fully convolutional neural network (Fully Convolutional Network, FCN) [17], the fully connected layers were replaced by fully convolutional layers, which introduced a new stage of medical image segmentation. Ronneberger et al. proposed a U-Net network structure for medical image segmentation [18], which used the encoding-decoding structure to restore image detail information and won the championship in both the ISBI 2015 Cell Tracking Challenge and the Tooth Decay Detection Challenge. Yuan et al. proposed a new loss function based on the Jaccard distance [19] to achieve automatic segmentation of skin lesion images. Sarker et al. used Space Pyramid Pooling (SPP) to alleviate the impact of uneven gray distribution in the lesion area on segmentation performance [20]. Dash et al. [21] proposed that PsLSNet was used to segment psoriasis skin lesions, improved the original U-Net topology, and adopted various efficient training strategies, which accelerated the training speed and improved the segmentation accuracy. Based on the U-Net network, Azad et al. combined with the mechanisms of BConvLSTM [22] and dense convolution [23], the proposed BCDU-Net achieved a better segmentation effect on the skin lesion [24]. Heidari et al. [25] proposed a novel method called HiFormer, which effectively connects CNN and Transformer for medical image segmentation. Although all of the above methods are desirable for the segmentation of skin lesions, the boundaries of the segmented images are relatively rough, and a lot of image detail information is lost.

In order to solve the above problems, we designed an Attention-based Dual-path Feature Fusion Network (ADFFNet), using VGG16 as the feature extraction basic network. Firstly, in order to effectively restore the image details, a Boundary Refinement (BR) module based on spatial attention is designed in the Spatial path to encode the rich spatial information in the lower layers, filter out the irrelevant backgrounds, and repair the boundary of the target area. Then a Multi-scale Feature Selection (MFS) module is built in the context path, capturing the object and image context at multiple scales, while eliminating redundant background information and integrating more effective context information. Finally, in the output stage, a Dual-path Feature Fusion (DFF) module is designed to fuse semantic information and spatial information, suppress the response of irrelevant background area, and better restore the detailed information of the image, so as to further improve the accuracy of the lesion area segmentation. We evaluate on the ISIC 2018 dataset and the PH2 dataset. The experimental results show that the network model proposed in this paper has advanced segmentation performance.

The main contributions of this paper are summarized as follows:

1. A novel skin lesion segmentation network is proposed to solve the problem of difficult lesion area segmentation in an end-to-end way. Compared with the existing skin lesion region segmentation network, this network is special in that it proposes a Boundary Refinement (BR) module to solve the problem of edge detail loss.
2. A multi-scale feature selection module and a dual-path feature fusion module are proposed. The Multi-scale Feature Selection module (MFS) can focus the network’s attention on channels that are more effective for segmentation, the Dual-path Feature Fusion (DFF) module uses global semantic information to guide feature fusion and helps the image to recover the detailed information more accurately and achieve the effect of accurate prediction.
3. Experiments on two public datasets show that the network model proposed in this paper is superior to most advanced methods in performance.

The remainder of this paper is organized as follows. The second section briefly introduces the related work. The third section introduces the details of the proposed method. In the fourth section, experiments and results are elaborated. The fifth part makes a concluding statement.

## Related works

Spatial Information CNN encodes high-level semantic information through a series of downsampling operations, which enriches high-level features, but severely loses spatial information. In the semantic segmentation task, the low-level spatial information is critical to the accuracy of prediction. Currently, mainstream segmentation networks are dedicated to encoding various spatial information. Many networks use U-shaped structures to recover spatial information. U-Net [18] uses jump connections based on FCNs to improve segmentation accuracy, however, a complete U-shape requires huge computational cost, especially for high-resolution images. SegNet [26] records the index of each MaxPooling and directly uses the recorded index to recover pixels during the upsampling process. The Deeplabs [27–29] series of work introduced atrous convolution to reduce the loss of feature resolution in the encoding part and preserve the spatial detail information of the feature maps.

Contextual Information Semantic segmentation requires context information to generate accurate pixel classification results. Due to the limitation of the convolutional layer structure, the context information provided by FCN is insufficient and needs to be improved. Therefore, in recent years, various methods have been proposed to explore context dependence to obtain more accurate segmentation results. So far, there are two main types of context information aggregation [30]. (1) Pyramid-based method: PSPNet [31] uses a pyramid pooling module to regularly aggregate regional or global context information. Deeplabv2 [27] uses the “ASPP” module to capture the context information of different receiving domains. DeepLabv3 [28] designed the “ASPP” module with a global average pooling to capture the global context information of the image. (2) The method based on self-similarity [32]: DANet [33], OCNet [34] fuse similar features at any scale from a global perspective. CCNet [35] captures contextual information from remote dependencies more effectively through horizontal and vertical cross-focus modules. In addition, EncNet [36] and DFN [37] added global pooling to encode the global context.

Attention Mechanism In recent years, attention mechanisms have been widely used in object detection and semantic segmentation tasks. For segmentation tasks, the attention mechanism is an extremely effective tool, it will strengthen the most informative feature expression while suppressing those less useful feature expressions and can guide the feedforward neural network to correct the output results. Attention to scale [38] uses the attention model to train and obtain the weight of each scale feature information and then fuses according to the weight. SENet [39] provides an effective, lightweight gating mechanism that comes from the calibration feature map through channel attention. Inspired by [40], some methods such as EncNet [36], BiseNet [40], etc. all use the channel attention mechanism to achieve the SOTA effect. DFN [37] extracts global semantic information as an attention vector to modify the output feature map. DANet [33] introduces a dual attention mechanism to obtain contextual relations. The so-called dual attention refers to the attention mechanism for channels and spaces.

Based on the development of the above-mentioned image semantic segmentation, the segmentation technology of skin lesions has also developed rapidly. Bi et al. [41] proposed an end-to-end multi-stage Fully Convolutional Network (mFCN) method for training and prediction of skin lesion segmentation, where they used a parallel integration method to combine the outputs of every stage. Masni et al. [42] proposed a new full-resolution convolutional network (FrCN) to segment skin lesions, which can generate complete spatial resolution features for each pixel of the input dermoscopic image, thereby improving the performance of pixel segmentation. Sarker et al. [20] proposed SLSDeep, which combines skip-connections, dilated residuals, and pyramid pooling, which expressed as an encoder-decoder structure. Their optimization function combines Negative Log Likelihood (NLL) and End Point Error (EPE) to accurately segment the melanoma regions. Esfahani et al. [43] introduce a Dense Pooling Fully Convolutional Network (DPFCN), using a new dense pooling layer to segment skin lesion regions.

## Method

In this section, we will introduce the proposed network in detail. The first is the overall network framework. Then is the boundary refinement module for the spatial path, the multi-scale feature selection module for context path, and the dual-path feature fusion module. Finally, the multivariate loss function is introduced.

### ADFFNet architecture

**Fig. 2:**
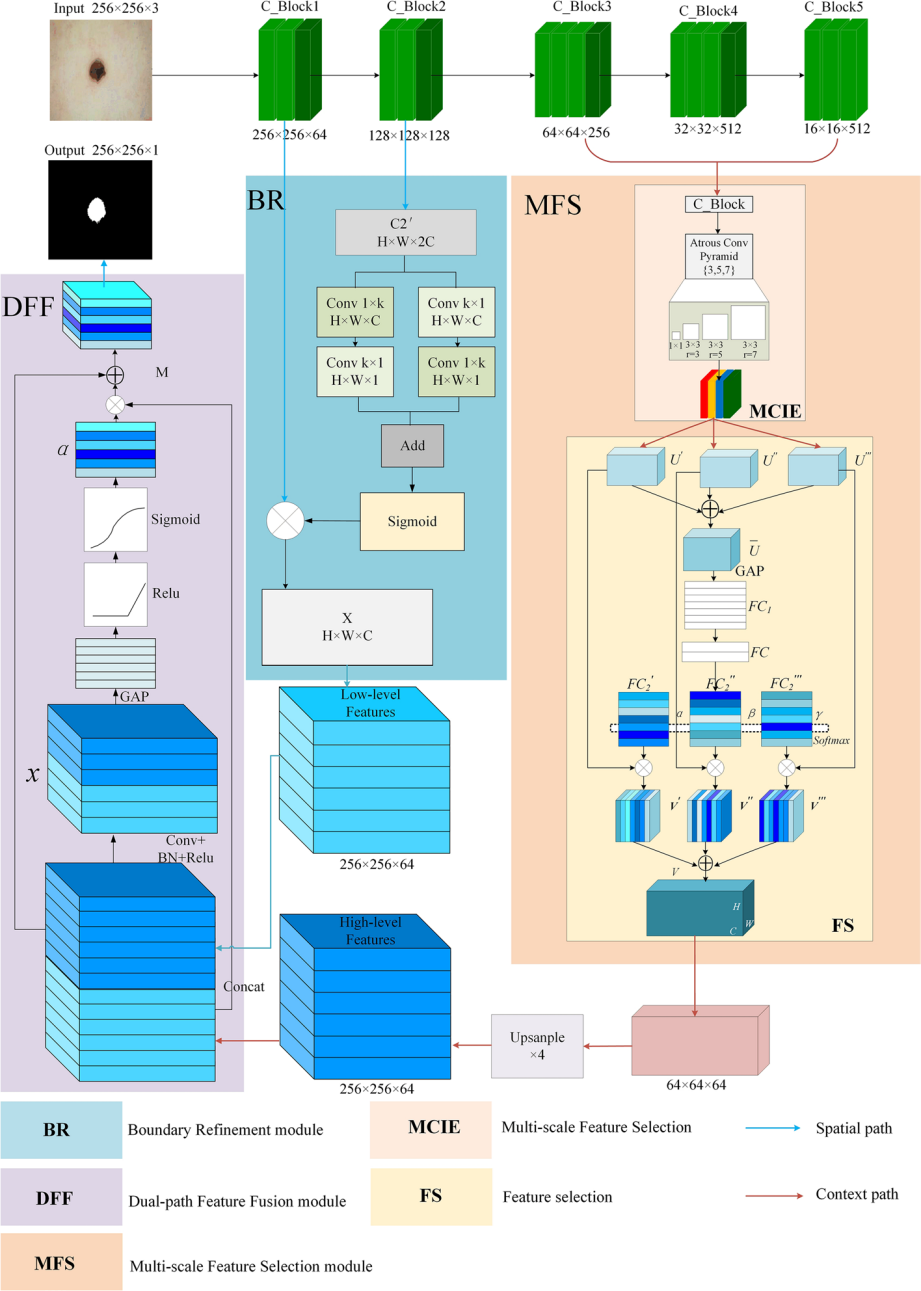
The architecture of ADFFNet module

In this section, we will introduce an overall framework named as Attention-based Dual-path Feature Fusion Network (ADFFNet). The overall network model structure is shown in Fig. 2. We use the classic VGG16 model as the network backbone, which has five basic convolutional blocks. Each convolutional block is composed of a different number of $3 \times 3$ convolutional layers and a maximum pooling layer. By halving the size of the feature map of each convolutional block, the number of filters is doubled to keep the time complexity of each layer unchanged. And a spatial path and a context path are created in the lower and upper layers of the network, respectively, so that the network can extract more spatial information in the lower layer features and obtain more context information in the upper layer features.

First of all, in the spatial path, we use the boundary refinement module to enhance the extraction of the relevant information of the edge of the lesion area, which can effectively solve the problem of the rough boundary segmentation of other lesion area segmentation networks and correct the final semantic segmentation result. Secondly, in the context path, the multi-scale feature selection module innovatively considers the enhancement and compression of effective information and redundant information in the multi-scale context, so that the network can achieve a better prediction and segmentation effect. Finally, the dual-path feature fusion module uses the attention mechanism to make the network pay more attention to the lesion foreground area of the skin melanoma image during the fusion stage, and output a more refined segmentation result.

### Boundary refinement module

**Fig. 3:**
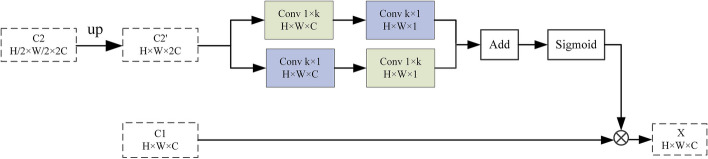
Structure of boundary refinement module

Pigmented skin lesions have irregular shapes and blurred boundaries, making it difficult to finely segment the boundaries. However, in the task of segmentation of the lesion area, the segmentation network needs to obtain the detailed boundary of the lesion. We find abundant spatial information exists in the low-level network, especially the details of the image boundary. Therefore, a boundary refinement module is designed in the spatial path at the low-level of the network to focus spatial attention on the target foreground region, which is helpful for fine segmentation of the boundary. As shown in Fig. 3, we represent the output feature maps of C_Block1 and C_Block2 as *C*1 and *C*2 respectively. We believe that *C*1 has more texture detail information, but also contains a lot of background interference information, while *C*2 has more semantic information, so we process the feature map of *C*2. $C2'$ is obtained by upsampling C2, using the long-strip kernel, can pay more attention to a certain area to avoid the introduction of irrelevant information brought by the traditional convolution kernel, and make the output feature map pay more attention to the image foreground. As shown in Eqs. (1) and (2), to increase the receiving field, obtain global information without increasing parameters, we use two convolutional layers, one kernel is 1$\times$k, and another kernel is k$\times$1 for feature map $C2'$. Then, using the Sigmoid operation to obtain the attention map *A* as shown in Eq. (3). As shown in Eq. (4), the final output *X* of the BR module is obtained by weighting *C*1 with *A*.

1 $$\begin{aligned} X_1=Conv_{k\times 1}(Conv_{1\times k}(C2',W_1^{1}),W_1^{2}) \end{aligned}$$

2 $$\begin{aligned} X_2=Conv_{1\times k}(Conv_{k\times 1}(C2',W_2^{1}),W_2^{2}) \end{aligned}$$

3 $$\begin{aligned} A=Sigmoid(X_1+X_2) \end{aligned}$$

4 $$\begin{aligned} X=A\cdot C1 \end{aligned}$$

Where *W* refers to the parameters of the convolution kernel, $Conv_{1\times k}$ and $Conv_{k\times 1}$ refers to the convolution layer of $1\times k\times C$ and $k\times 1\times 1$ respectively. In our experiments, we set $k=3$.

### Multi-scale feature selection module

The multi-level feature maps not only have rich context-aware information, but also contain some redundant information that is useless for segmentation. Therefore, this paper uses context-aware pyramid features to extract multi-scale context information at the high level of the network, and to filter the features, adaptively assigning more network attention to the feature channels that more effective for segmentation of the lesion area, thereby improving the skin segmentation effect of the lesion area.

#### Multi-scale context information extraction

Context information is very important for image semantic segmentation. Existing CNN models often extract object features by stacking multiple convolutions and pooling layers. Due to the unevenness of contrast or colour of the skin lesion area, an effective skin lesion segmentation method should be able to segment the lesion area according to the context information of its area. The segmentation of the ambiguous skin lesion area depends on the segmentation of the significant lesion area. However, the size and shape of the lesion area are very different, so the single-scale context information from the input image cannot effectively guide the feature extraction of different scales, and the single-size convolution kernel is difficult to effectively extract the multi-scale information of the lesion area. Therefore, inspired by literature [27], we design a multi-scale context information extraction module based on atrous convolution.

**Fig. 4:**
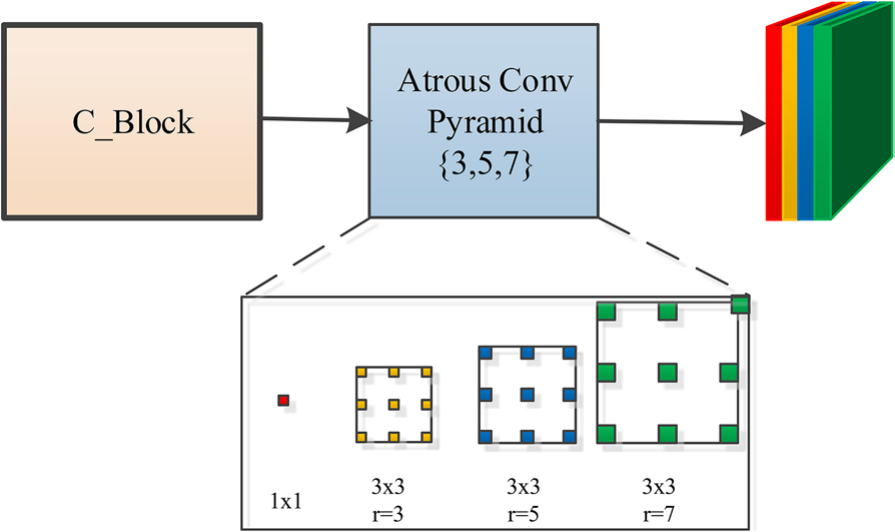
Structure of multi-scale context information extraction

For the skin lesion ISIC 2018 dataset, it is not appropriate to use an excessively large void rate. Too large dilated rate will result in too much useless information of the extracted features and loss of useful information, which will lead to unclear edges and missed segmentation of the segmented lesions. Therefore, we use small dilated rate of 3, 5, 7. As shown in Fig. 4, it consists of parallel 1$\times$1 convolution and three 3$\times$3 dilated convolutions with the dilation rate of 3, 5, 7 respectively. The original information of the image is preserved through the 1$\times$1 convolution, and the semantic information is extracted by the dilated convolution. Finally, all the feature maps are channel spliced. Small-sized convolution kernels are more interested in the local details of skin lesions, while large-sized convolution kernels are more interested in the contour information of skin lesions. We use multi-scale dilated convolution to not only extract better local details, and have a better characterization of the contour of the skin lesion area.

### Feature selection based on attention mechanism

**Fig. 5:**
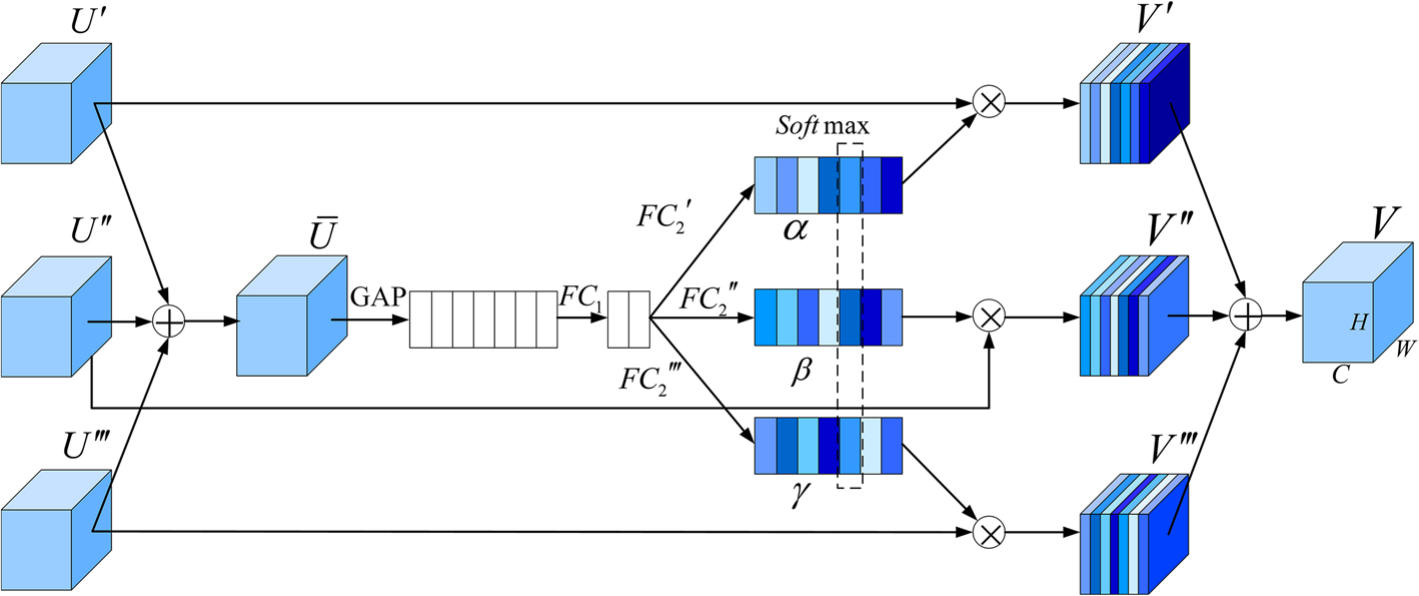
Structure of feature selection

In order to further filter the multi-scale semantic information captured by the multi-scale context information extraction module, inspired by SKNet [20], this paper designs a feature selection structure. During the training process, the feature selection structure can assign a larger weight to the channel that contributes the most to the segmentation of the lesion area and adaptively select the most suitable receptive field and feature size to achieve accurate prediction. Figure 5 is the detailed feature selection structure. Among them, $U'$, $U''$, $U'''\in R^{H\times W\times C}$ respectively represents the feature maps obtained by the convolutional blocks of $C_{Block3}$, $C_{Block4}$, $C_{Block5}$ and after multi-scale context information extraction. As shown in Eq. (5), $\bar{U}$ is obtained by summing element by element and integrating the information of multiple branches:

5 $$\begin{aligned} \bar{U}=U'+U''+U''' \end{aligned}$$

Then, the attention feature vectors $\alpha$, $\beta$, $\gamma$ are obtained by using global average pooling and two fully connected layers and a Softmax layer. The final output feature map is $V=[V_1,V_2,...,V_c]$, where *c* represents the number of channels. The $i(0<i<c)$ channel $V_i$ is:

6 $$\begin{aligned} V_i=\alpha _iU'+ \beta _iU''+ \gamma _iU''' \end{aligned}$$

where $\alpha _i$, $\beta _i$, $\gamma _i$ is the variable of vector $\alpha$, $\beta$, $\gamma$, and satisfy Eq. (7):

7 $$\begin{aligned} \alpha _i+ \beta _i+ \gamma _i=1 \end{aligned}$$

### Dual-path feature fusion module

Skin melanoma images usually contain a lot of foregrounds and complex background information, such as hair occlusion. In our proposed model, the low-level feature map obtained through the spatial path has a lot of detailed information but contains a lot of useless background information, the high-level feature map obtained through the context path contains rich semantic information but lacks detailed information. But in semantic segmentation networks, simple feature fusion, such as pixel sum and channel splicing, often ignores the inconsistency between semantic information and detailed features. Therefore, we design a Dual-path Feature Fusion (DFF) module based on the attention mechanism, which is used to pay more attention to the lesion foreground area of the skin melanoma image.

**Fig. 6:**
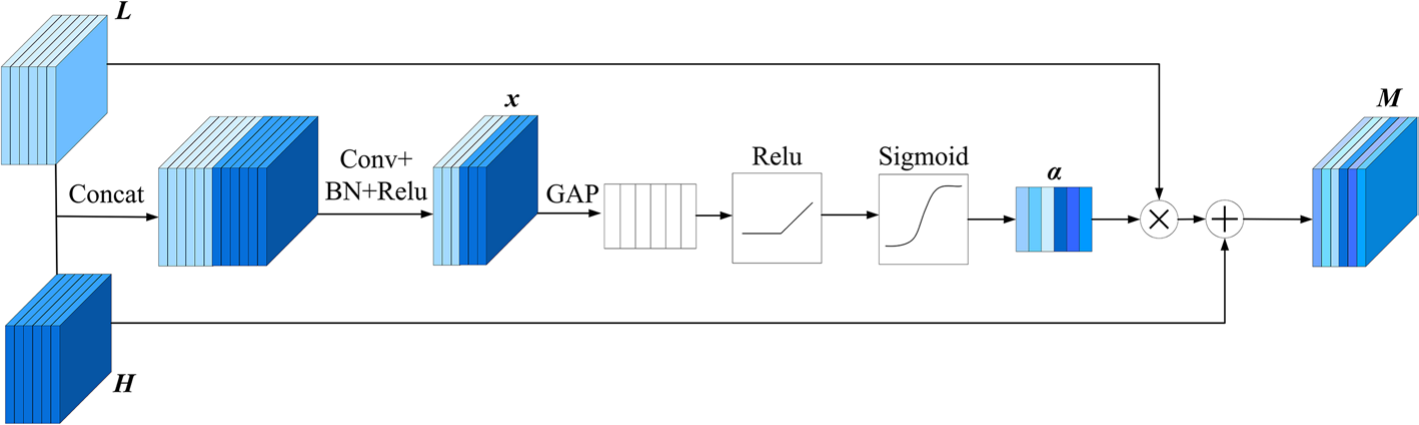
Structure of dual-path feature fusion module

As shown in Fig. 6, $H\in R^{H\times W\times C}$ represents the high-level semantic feature from the context path, and $L\in R^{H\times W\times C}$ represents the low-level detailed feature from the spatial path. Firstly, the high-level semantic features and the low-level detailed features are connected in series, batch normalization is used to balance the data distribution of the features, and the feature vectors *x* is obtained through the Relu activation function, as shown in Eq. (8).

8 $$\begin{aligned} x=Relu(BN(Conv(Concat(H,L)))) \end{aligned}$$

Where, *Conv* denotes the convolution operation, and *Concat* denotes the operation of concatenating the channels of *H* and *L*.

The mathematical expression of *ReLU* function is as follows:

9 $$\begin{aligned} f(x)=max(0,x) \end{aligned}$$

*x* represents the input value.

The mathematical expression of *BN* function is as follows:

10 $$\begin{aligned} BN(x)= \gamma \frac{x-\mu }{\sqrt{\delta ^2 + \epsilon }} + \beta \end{aligned}$$

Where, *x* represents the input data. $\mu$ is the mean of the input data over the batch. $\delta ^2$ is the variance of the input data over the batch. $\gamma$ is the scaling factor. $\beta$ is the shifting factor. $\epsilon$ is a small constant. The purpose of the *BN* function is to ensure that the input to each layer in the network maintains a certain mean and variance, thereby accelerating network training and improving model stability.

Secondly, as shown in Eq. (11), for the feature vector *x*, using Global Average Pooling (GAP) to extract the global average vector, and the attention weight vector *a* is obtained through the Relu activation function and the Sigmoid operation in turn.

11 $$\begin{aligned} a=Sigmoid(Relu(GAP(x))) \end{aligned}$$

The mathematical expression of *Sigmoid* function is as follows:

12 $$\begin{aligned} f(x)=\frac{1}{1+ e^{-x}} \end{aligned}$$

As shown in Eq. (13), use the attention weight vector *a* to weight *L*, the final output $M\in R^{H\times W\times C}$ is obtained by adding *H* with the weighted feature map.

13 $$\begin{aligned} M=Attention(a,L)+H \end{aligned}$$

*Attention*(*a*, *L*) represents the calculation of the Attention mechanism, and its mathematical expression is as follows:

14 $$\begin{aligned} A(a,L)=Sigmoid(MLP(Avgpool(a,L))+MLP(Maxpool(a,L))) \end{aligned}$$

The DFF module uses advanced features to provide semantic information to guide feature fusion, so that the network can correctly focus on the foreground information of the lesion area in the skin melanoma image, thereby generate more discriminative fusion features and improve segmentation accuracy.

###  Multivariate loss function

In machine learning and mathematical optimization, the loss function can be regarded as an evaluation of the prediction result. Usually, we use the cross-entropy loss to evaluate the segmentation effect. However, for the segmentation of the lesion area, there are problems of imbalance between the foreground and background categories and the difficulty of separating the boundary. The cross-entropy loss cannot be effective for the above-mentioned problems, which can easily lead to the model being more inclined to the category with a larger pixel ratio during the training process, making the model difficult to be fully trained, and the segmentation effect for the small category is poor. Therefore, we design the multivariate loss function.

Semantic boundary For the segmentation of the lesion area, the difficulty lies in the accurate segmentation of the boundary. Inspired by edge detection algorithms, the traditional edge detection operators can better strengthen the region boundary segmentation. Thus, in this article, the second-order edge detection Laplace operator is used to obtain the boundary information of the ground-truth label and the predicted semantic segmentation mask.

We can find a discrete convolution kernel $K_{Laplace}$, which is equivalent to the Laplacian operator, as shown in Eq. (15).

15 $$\begin{aligned} K_{Laplace} =\left[ \begin{array}{ccc} -1 &{} -1 &{} -1 \\ -1 &{} 8 &{} -1 \\ -1 &{} -1 &{} -1 \end{array}\right] \end{aligned}$$

As shown in Eq. (12), we use convolution calculation to obtain the Laplace edge detection map $\nabla T(x,y)\in R^{H\times W\times C}$ from the segmentation ground-truth map $T(x,y)\in R^{H\times W\times C}$.

16 $$\begin{aligned} \nabla T(x,y)= Conv(T(x,y),K_{Laplace}) \end{aligned}$$

17 $$\begin{aligned} E_t= Relu(Tahn(\nabla T)) \end{aligned}$$

**Fig. 7:**
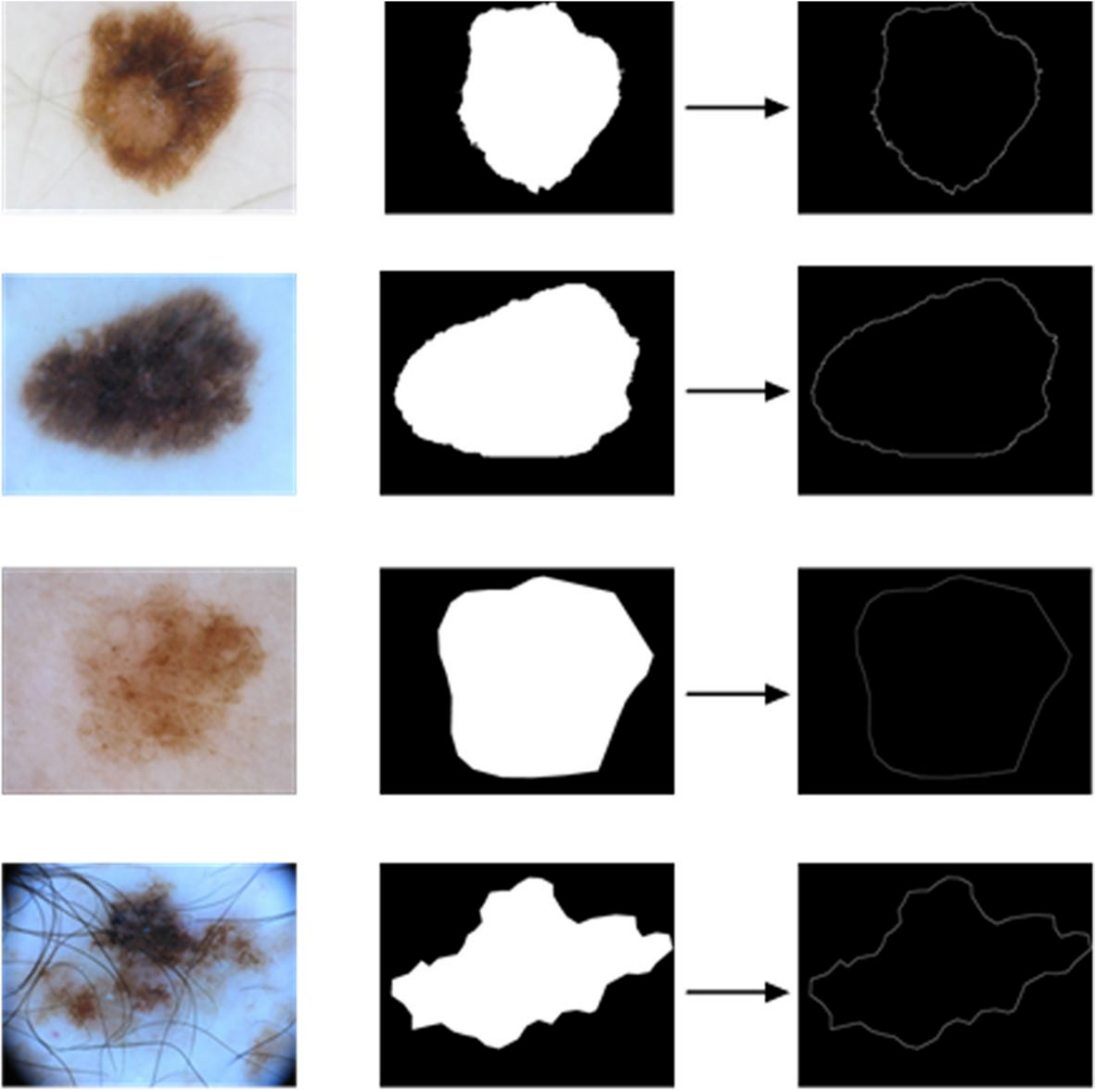
Some visualization examples. (a), (b), and (c) are original image, segmentation ground-truth label, and inferred true segmentation boundary map, respectively

To get a clearer map, as shown in Eq. (17), we first use the Tahn function to transform the value of the element in $\nabla T$ to [-1,1]. Then the Relu function is used to truncate, only the positive activation is taken, and finally the true segmentation boundary label $E_t\in R^{H\times W\times C}$ is obtained, as shown in Fig. 7. After the Dual-path Feature Fusion (DFF) module, we can get the predicted segmentation map $P(x,y)\in R^{H\times W\times C}$ , which is then treated with Eqs. (12) and (13) to get the predicted segmentation boundary map $E_p\in R^{H\times W\times C}$.

Boundary-oriented loss function. Due to the sparseness of pixels around the boundary, the predicted semantic boundary has a high missing rate. In order to reduce this effect, we define $W_{Laplace}$ and the boundary-oriented loss function $L_E$ to help locate the detailed boundary. $W_{Laplace}$ is boundary weight, which represents the percentage of boundary pixels to all pixels in the segmentation ground-truth label, and its value is between [0, 1], as shown in Eq. (18).

18 $$\begin{aligned} W_{Laplace}= \sum \limits _{i=1}^{N} E_t^{i} / \sum \limits _{i=1}^{N} T^{i} \end{aligned}$$

Where $E_t$ represents the true segmentation boundary map, *T* represents the segmentation ground-truth label, and *N* means the total number of pixels. Thus, the boundary-oriented loss function $L_E$ is finally formulated as below:

19 $$\begin{aligned} L_E= - \frac{1}{N} \sum \limits _{i=1}^{N}\left[ W_{Laplace}E_t^{i}log E_p^{i}+ (1- W_{Laplace})(1-E_t^{i})log(1- E_p^{i}))\right] \end{aligned}$$

Where $E_p$ represents the predicted segmentation boundary map.

Multivariate loss function The multivariate loss function can pay attention to the pixel-level classification accuracy of the entire image and the boundary at the same time. It can also make the model training more stable, and effectively overcome the imbalance between positive and negative samples, thereby obtaining more accurate segmentation results.

The multivariate loss function $L_{Total}$ is shown in Eq. (20), which consists of $L_E$ for boundary segmentation, a cross-entropy loss $L_{BCE}$ in Eq. (21) and a dice coefficient loss $L_{Dice}$ in Eq. (22) for semantic segmentation.

20 $$\begin{aligned} L_{Total}=\lambda _1L_E + \lambda _2L_{BCE} + \lambda _3L_{Dice} \end{aligned}$$

21 $$\begin{aligned} L_{BCE}=- \frac{1}{N} \sum \limits _{i=1}^{N}\left[ T^{i}log P^{i}+ (1-T^{i})log(1- P^{i})\right] \end{aligned}$$

22 $$\begin{aligned} L_{Dice}=1- \frac{\sum _{i=1}^{N} P^{i}T^{i}+\varepsilon }{\sum _{i=1}^{N}P^{i}+T^{i}+\varepsilon }- \frac{\sum _{i=1}^{N} (1-P^{i})(1-T^{i})+\varepsilon }{\sum _{i=1}^{N}2-P^{i}-T^{i}+\varepsilon } \end{aligned}$$

Where, $\lambda _1$ , $\lambda _2$ and $\lambda _3$ are hyperparameter. In our experiments, we set them as 0.2, 1, and 1 respectively based on experience, *P* represents the predicted segmentation map and *T* represents the segmentation ground-truth map. $\varepsilon$ is a settable value, which is used to prevent overfitting. In our experiments, we set it as 1.

## Experiments and analysis

### Dataset

The ISIC 2018 dataset [44] was published by the International Skin Imaging Collaboration (ISIC) as a large-scale dataset of dermoscopy images. The dataset comes from the challenges of lesion segmentation, dermoscopy feature detection, and disease classification, including 7 types of skin diseases such as melanoma and nodular melanoma. It includes 2594 images, we used 1815 images for training, 259 images for validation, and 520 images for testing. The images in the original dataset provided by ISIC 2018 have different resolutions. So, we pre-process the input image and resize the images to 256$\times$256 pixels. To address the issue of a limited number of images in the dataset and the potential problem of model overfitting during training, we employed the generative adversarial networks (GANs) [45] method to augment the dataset.

The PH2 dataset [46] was published by Mendonça et al. in 2013. There is a total of 200 dermoscopy images, including 160 pictures of moles and 40 pictures of melanoma. Each image contains only one lesion area, and the labeling of the segmented areas is continuous. The network is only trained on the ISIC 2018 dataset and tested directly on the PH2 dataset. All the images in the original dataset provided by PH2 are 8-bit RGB images with 768$\times$560 pixels resolution. We pre-process the input image and resize the images to 256$\times$256 pixels.

### Evaluation metrics

We use general segmentation evaluation metrics to evaluate the segmentation performance of our proposed network, including Accuracy (AC), Sensitivity (SE), Specificity (SP), Precision (PC), Jaccard similarity (JS), and F1 score. Among all the indicators, JS is generally considered to be the most important criterion for segmentation. The criteria are defined as below:

23 $$\begin{aligned}{} & {} AC= \frac{N_{TP}+N_{TN}}{N_{TP}+N_{FP}+N_{FN}+N_{TN}},\nonumber \\{} & {} SE= \frac{N_{TP}+N_{TN}}{N_{TP}+N_{FN}},\ SP= \frac{N_{TN}}{N_{TP}+N_{FP}},\nonumber \\{} & {} PC= \frac{N_{TP}}{N_{TP}+N_{FP}},\nonumber \\{} & {} F1= \frac{2 \times PC\times SE}{PC+SE},\nonumber \\{} & {} JS=\frac{N_{TP}}{N_{TP}+N_{FP}+N_{FN}} \end{aligned}$$

where $N_{TP}$, $N_{TN}$, $N_{FP}$, $N_{FN}$ denote the number of true positive, true negative, false positive, and false negative, respectively, and they are all defined on the pixel level. A lesion pixel is considered as a true positive if its prediction is lesion; otherwise, it is regarded as a false negative. A non-lesion pixel is considered as a true negative if its prediction is non-lesion; otherwise, it is regarded as a false positive.

### Implementation details

The experiments were carried out in a hardware environment with the GPU model of NVIDIA GTX1080Ti. The deep learning framework used is Keras, using CuDNN V7 and Cuda9.1 version. During the training process, we randomly initialize the model weights and bias terms to obey the standard Gaussian distribution and use the Adam optimizer to optimize the network parameters. The Adam function parameters used in Keras are $L_r$(Learning rate)=0.0001, $beta\_1$=0.9, $beta\_2$=0.999, $decay=1\times 10^{-4}$. The model is trained a total of 60 batches, and the size of each batch is set to 8. When the loss of the validation set exceeds 10 epochs and does not decrease, the training is stopped and the model is considered to be optimal.

**Fig. 8:**
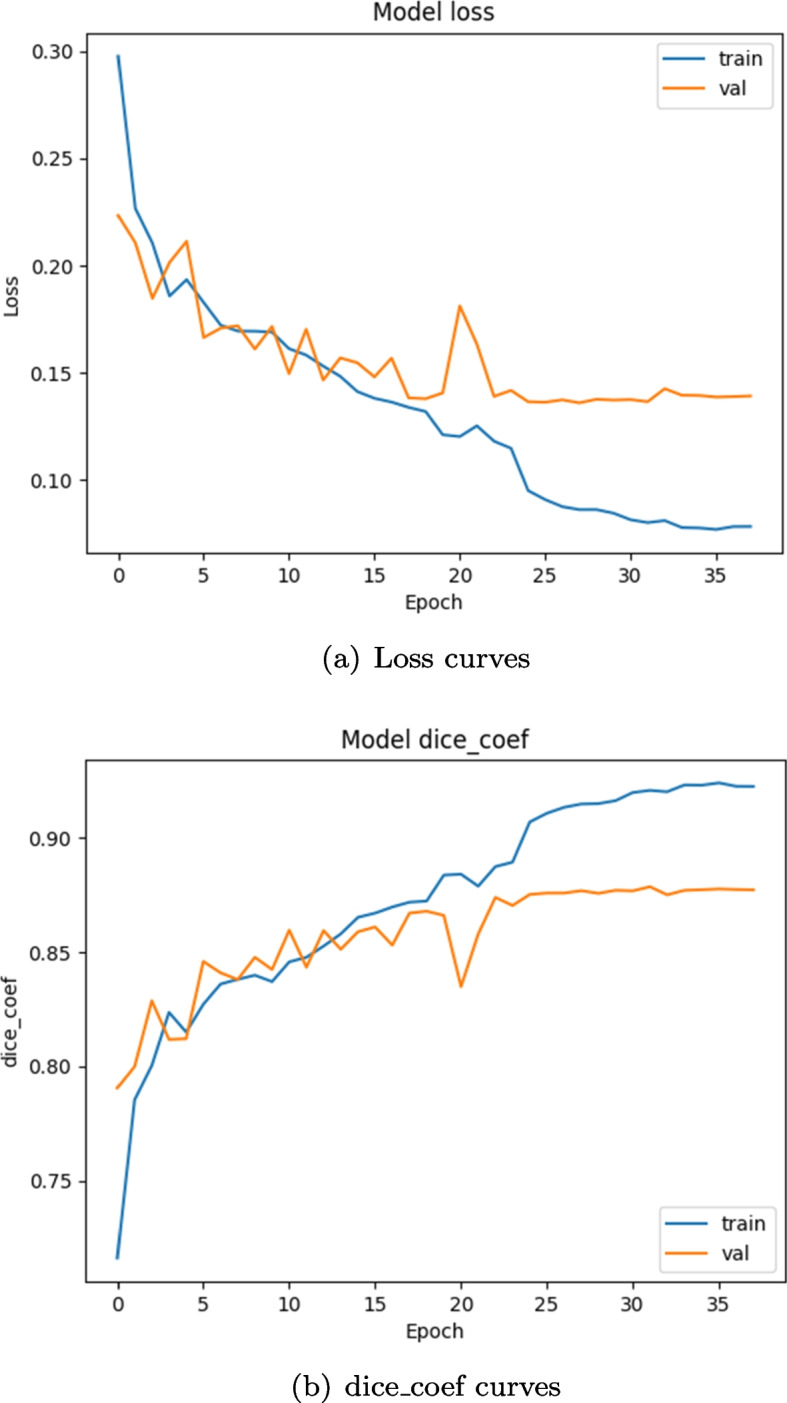
Visualization curve in the training process of the model. **a** Visualization of loss, **b** Visualization of dice coefficient

During the training process, we chose the dice coefficient as the evaluation index. The training is stopped after 60 epochs. The visual loss curve and dice_coef index are shown in Fig. 8. After 35 epochs, the model begins to converge and eventually tends to be stable on the training set and verification set.

### Ablation experiments

In order to verify the effectiveness of the modules designed in this paper, it is necessary to perform ablation experiments on the ISIC 2018 dataset. The main comparison index selected is JS. The indicator comparison is shown in Table 1, the experiment of No. 1 is the result of the U-Net model without any module. It can be seen that the Multi-scale Context Information Extraction (MCIE) has the greatest effect on the model performance improvement. The reason is that the multi-dilation rate atrous convolution is combined with the multi-scale context information, which can accurately segment melanomas of different sizes and shapes. Similarly, the Boundary Refinement (BR) module, the Feature Selection (FS) structure, and the Dual-path Feature Fusion (DFF) module can greatly improve the performance of the model, which can effectively improve the segmentation accuracy of melanoma.

**Table 1:** Ablation experiments using different component combinations

Number	BR	MCIE	FS	DFF	JS
1					0.590
2	$\surd$				0.635
3		$\surd$			0.628
4			$\surd$		0.671
5				$\surd$	0.649
6	$\surd$	$\surd$			0.717
7	$\surd$	$\surd$	$\surd$		0.755
8	$\surd$	$\surd$	$\surd$	$\surd$	0.783

### Results on dataset

We conducted training and testing on the ISIC 2018 dataset to prove the effectiveness of the proposed network, and only tested on the PH2 dataset, verifying the generalization ability of the proposed network. Tables 2 and 3 are the comparison of indicators between the mainstream methods and the proposed method on the ISIC 2018 dataset and PH2 dataset respectively. The backbone is U-Net.

**Table 2:** Performance evaluation of different segmentation methods on ISIC2018 dataset

Methods	F1-score	SE	SP	AC	PC	JS
UNet [18]	0.742	0.708	0.964	0.890	0.779	0.590
Attention UNet [47]	0.750	0.717	0.967	0.897	0.787	0.600
R2UNet [48]	0.766	0.792	0.928	0.880	0.741	0.620
FCN [17]	0.852	0.837	0.966	0.938	0.868	0.742
UNet++ [49]	0.856	0.817	0.975	0.942	0.900	0.748
BCDUNet [24]	0.851	0.785	0.982	0.937	0.928	0.740
HiFormeS [25]	0.883	0.928	0.911	0.918	0.848	0.795
Ours(VGG)	0.873	0.827	0.982	0.950	0.924	0.774
Ours(ResNet101)	0.890	0.933	0.918	0.927	0.880	0.819

**Table 3:** Performance evaluation of different segmentation methods on PH2 dataset

Methods	F1-score	SE	SP	AC	PC	JS
UNet [18]	0.789	0.781	0.851	0.859	0.787	0.651
Attention UNet [47]	0.823	0.796	0.934	0.889	0.852	0.700
R2UNet [48]	0.834	0.812	0.923	0.896	0.857	0.715
BCDUNet [24]	0.866	0.864	0.938	0.914	0.869	0.764
HiFormeS [25]	0.922	0.982	0.903	0.937	0.871	0.856
Ours(VGG)	0.897	0.893	0.954	0.934	0.902	0.814
Ours(ResNet101)	0.925	0.954	0.922	0.945	0.915	0.872

As can be seen from Table 2,on the ISIC 2108 dataset, the F1-score , SE and JS indexes of the method in this paper have improved by 0.7%, 0.5% and 2.4% respectively, compared with the highest value of all the comparison methods. Among the indicators, AC and SP reached the highest, while PC was close to the best results. As shown in Table 3, the F1-score, SP, AC, PC and JS indexes of this method in PH2 dataset have improved 0.3%, 1.6%, 0.8%, 4.4%, and 1.6% respectively. Although SE did not reach the maximum, they remained relatively stable and the overall performance of the model was satisfactory. It indicated that the proposed model has better generalization ability.

**Fig. 9:**
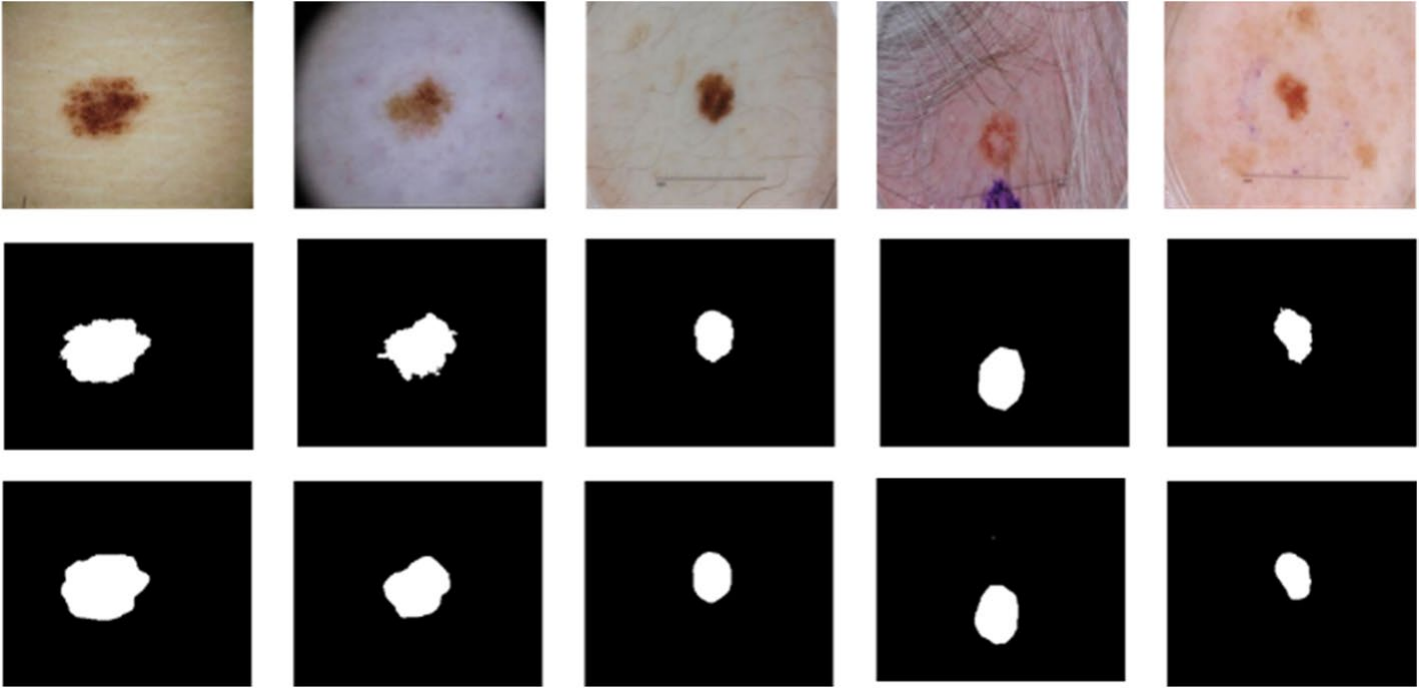
Segmentation results on ISIC 2018 dataset. Top: input images; Middle: ground-truth labels; Bottom: prediction images

**Fig. 10:**
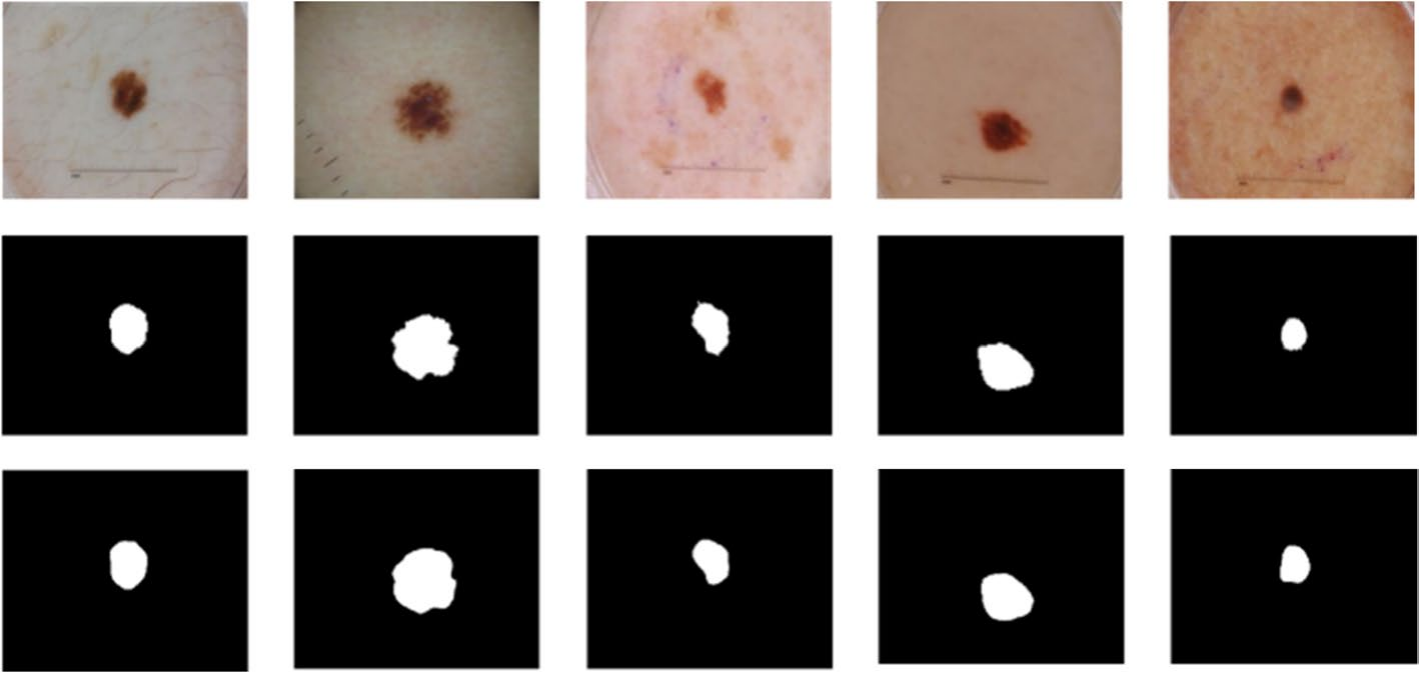
Segmentation results on PH2 dataset. Top: input images; Middle: ground-truth labels; Bottom: prediction images

**Fig. 11:**
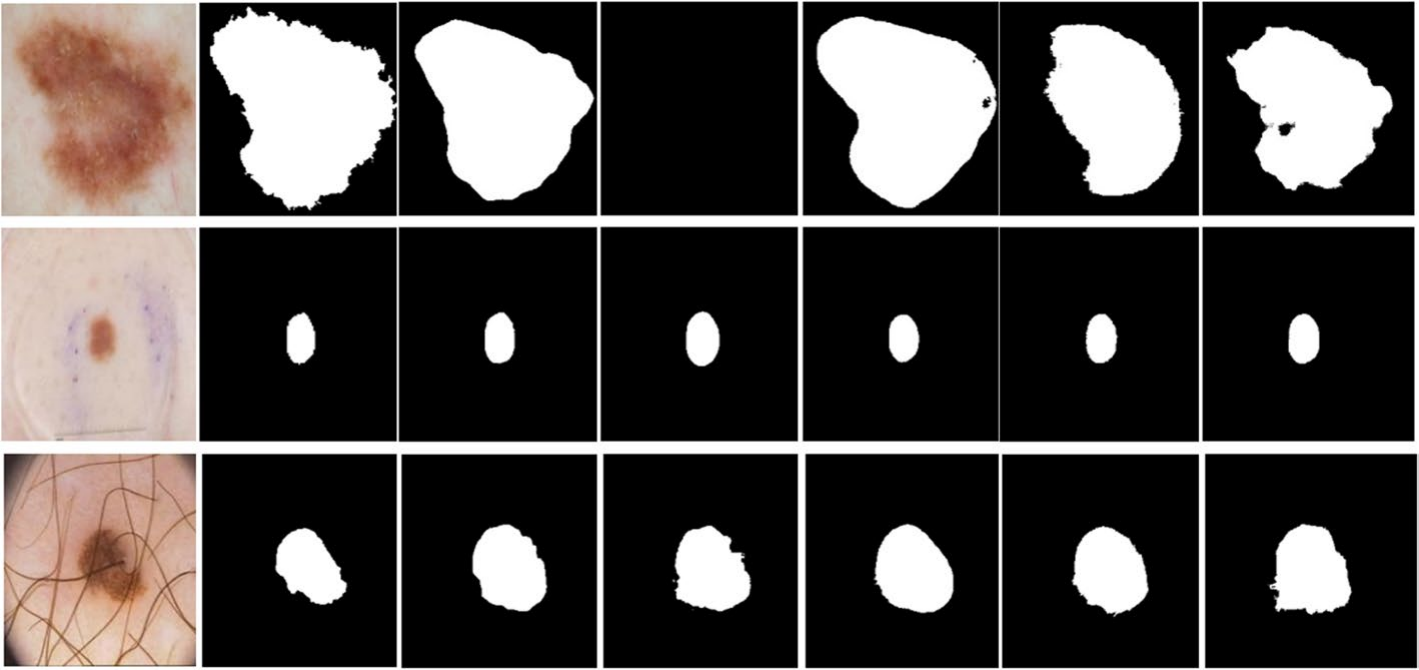
Segmentation results comparison of ADFFNet with other methods. From left to right: Images, Ground truth, Ours, ATTU-Net, BCDU-Net, R2U-Net and U-Net

Figures 9 and 10 are the comparison diagrams of the prediction results of our method on the ISIC 2018 and PH2 datasets with the true labels. In some challenging cases of melanoma lesions, including cases with complex background and low contrast, our method has achieved satisfactory results. Figure 11 provides a comparison between our method and other advanced methods. In summary, our method has achieved excellent results in the segmentation of skin melanoma on the ISIC 2018 and PH2 datasets.

### Performance analysis

We compared the complexity of our ADFFNet with the complexities of other methods. We used input feature maps of size $256 \times 256$ to evaluate their complexity during inference, and measured the training parameters, computation complexity (measured by the number of FLOPs), and inference time (measured by average inference time for each image). The test results are shown in Table 4, our proposed model has the smallest FLOPs. For FLOPs, parameters and inference time, our proposed model ensures that these three indicators can achieve relatively favorable results when compared to other algorithms while maintaining the highest accuracy. According to Table 4, our ADFFNet has optimal efficiency from a comprehensive point of view.

**Table 4:** Complexity comparison. The numbers are obtained on a single NVIDIA GTX1080Ti GPU. All the numbers are the smaller the better

Methods	FLOPs	Parameters(M)	Time(ms)
U-Net [18]	44.4G	10.1	8.4
Attention-UNet [47]	47.4G	22.2	8.8
R2U-Net [48]	50.9G	25.4	18.3
BCDU-Net [24]	39.8G	20.7	28.8
HiFormeS [25]	17.02G	31.5	17.1
Ours(VGG)	32.4G	16.2	16.3
Ours(ResNet101)	17.18G	46.2	17.1

**Fig. 12:**
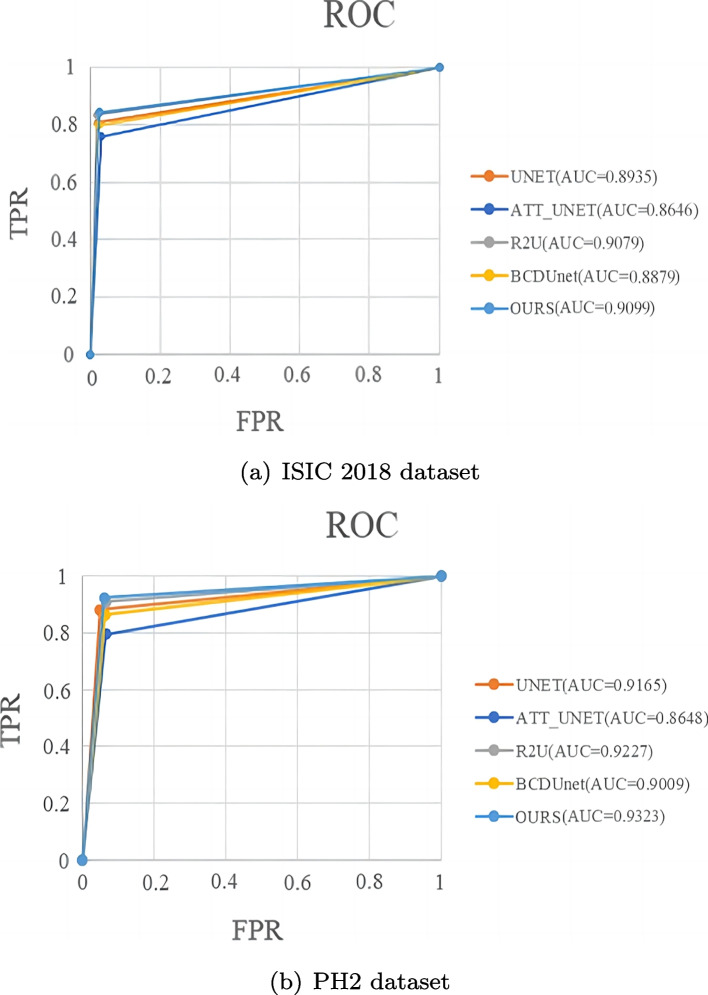
Comparison of the ROC curves for two datasets

To evaluate the overall performance of the proposed network on the ISIC 2018 and PH2 datasets, the visual Receiver Operating Characteristic (ROC) curve. ROC curve is a graph composed of True Positive Rate (TPR) and False Positive Rate (FPR). Area Under Curve (AUC) is the area under the ROC curve, which can measure the ability of the network to segment input images. The better the model classification, the greater the corresponding AUC. Figure 12 shows the ROC curve of the different networks, our method shows better overall performance on both datasets, with AUC reaching 0.9099 and 0.9323 respectively.

## Conclusion

In this paper, we propose an Attention-based Dual-path Feature Fusion Network (ADFFNet) for automatic segmentation of skin lesions. Considering the feature expression at different stages of the network, for the features of the advanced stage of the network, in the context path, a Multi-scale Feature Selection module (MSF) is proposed, which can capture different levels of semantic expression and adaptively adjust the size of the receptive field, and assign a larger weight to the channel that contributes the most to the segmentation of the lesion area to achieve the effect of accurate prediction. For the detailed features of the low-level stage of the network, in the spatial path, a Boundary Refinement (BR) module based on the attention mechanism is proposed to suppress irrelevant background and strengthen the edge information. In the output stage of the network, through the Dual-path Feature Fusion module (DFF), the high-level semantic information is used to guide the recovery of the low-level detail information to obtain better segmentation results. In addition, traditional edge detection operators are integrated to guide the network to learn more details about boundary positioning. Experimental results on the ISIC 2108 and PH2 datasets show that the proposed method is superior to the existing advanced methods in the segmentation task for melanoma, and has excellent generalization ability. In short, the method in this paper has strong feature extraction capabilities and accurate image semantic segmentation capabilities, and can accurately locate and segment skin lesions, thereby obtaining better performance in melanoma detection.

While the methodology presented in this study has exhibited commendable performance in the skin lesions, there are some limitations. Our research primarily gravitates toward the segmentation of skin lesions. Nevertheless, within the scope of practical clinical applications, there arises the necessity for lesion categorization and diagnosis. Consequently, forthcoming investigations may contemplate the amalgamation of our approach with lesion categorization and diagnosis, thereby engendering a more comprehensive analysis of cutaneous abnormalities.

## Data Availability

The datasets used or analyzed during the current study are available from the corresponding author on reasonable request.
